# Mutual Suppression of Proximal and Distal Axonal Spike Initiation Determines the Output Patterns of a Motor Neuron

**DOI:** 10.3389/fncel.2019.00477

**Published:** 2019-10-23

**Authors:** Nelly Daur, Yang Zhang, Farzan Nadim, Dirk Bucher

**Affiliations:** ^1^Federated Department of Biological Sciences, New Jersey Institute of Technology and Rutgers University-Newark, Newark, NJ, United States; ^2^Department of Mathematical Sciences, New Jersey Institute of Technology, Newark, NJ, United States

**Keywords:** ectopic spikes, dopamine, axon, neuromodulation, neural oscillations, central pattern generation, after-hyperpolarization

## Abstract

Axonal spike initiation at sites far from somatodendritic integration occurs in a range of systems, but its contribution to neuronal output activity is not well understood. We studied the interactions of distal and proximal spike initiation in an unmyelinated motor axon of the stomatogastric nervous system in the lobster, *Homarus americanus*. The peripheral axons of the pyloric dilator (PD) neurons generate tonic spiking in response to dopamine application. Centrally generated bursting activity and peripheral spike initiation had mutually suppressive effects. The two PD neurons and the electrically coupled oscillatory anterior burster (AB) neuron form the pacemaker ensemble of the pyloric central pattern generator, and antidromic invasion of central compartments by peripherally generated spikes caused spikelets in AB. Antidromic spikes suppressed burst generation in an activity-dependent manner: slower rhythms were diminished or completely disrupted, while fast rhythmic activity remained robust. Suppression of bursting was based on interference with the underlying slow wave oscillations in AB and PD, rather than a direct effect on spike initiation. A simplified multi-compartment circuit model of the pacemaker ensemble replicated this behavior. Antidromic activity disrupted slow wave oscillations by resetting the inward and outward current trajectories in each spike interval. Centrally generated bursting activity in turn suppressed peripheral spike initiation in an activity-dependent manner. Fast bursting eliminated peripheral spike initiation, while slower bursting allowed peripheral spike initiation to continue during the intervals between bursts. The suppression of peripheral spike initiation was associated with a small after-hyperpolarization in the sub-millivolt range. A realistic model of the PD axon replicated this behavior and showed that a sub-millivolt cumulative after-hyperpolarization across bursts was sufficient to eliminate peripheral spike initiation. This effect was based on the dynamic interaction between slow activity-dependent hyperpolarization caused by the Na^+^/K^+^-pump and inward rectification through the hyperpolarization-activated inward current, *I*_*h*_. These results demonstrate that interactions between different spike initiation sites based on spike propagation can shift the relative contributions of different types of activity in an activity-dependent manner. Therefore, distal axonal spike initiation can play an important role in shaping neural output, conditional on the relative level of centrally generated activity.

## Introduction

Canonically, action potentials (spikes) are initiated at a single site, usually the soma or proximal axon, as the result of integration of somatodendritic synaptic inputs or endogenous membrane oscillations. Spikes then propagate along the axon with high fidelity to distal presynaptic sites. However, it has long been known that some neurons have more than one initiation site and can generate activity from spatially separated signal integration (Calabrese and Kennedy, 1974; O’Shea, 1975; Vedel and Moulins, 1977; Moulins et al., 1979), or use dendritic spike initiation to amplify synaptic information transfer to the axon (Reyes, 2001; Canals et al., 2005). In addition, some neurons exhibit distal axonal spike initiation, not directly resulting from somatodendritic integration (Bucher and Goaillard, 2011; Debanne et al., 2011; Sasaki, 2013; Bucher, 2015; Rama et al., 2018). Such spike initiation is unequivocally “ectopic” when it occurs in abnormal places, which is a common phenomenon in a range of neuropathies associated with injury, demyelination, inflammation, or seizure activity (Stasheff et al., 1993; Pinault, 1995; Poliak and Peles, 2003; Ma and LaMotte, 2007; Krishnan et al., 2009; Connors and Ahmed, 2011; Hamada and Kole, 2015; Meacham et al., 2017). However, spike initiation in more distal axonal compartments can also occur under normal physiological conditions. For example, hippocampal interneurons and pyramidal cells generate such spikes in a range of different network states, which has been proposed to contribute to network oscillations and memory formation (Pinault, 1995; Avoli et al., 1998; Epsztein et al., 2010; Bahner et al., 2011; Connors and Ahmed, 2011; Sheffield et al., 2011; Dugladze et al., 2012; Bukalo et al., 2013; Sheffield et al., 2013; Buzsaki, 2015).

Distal axonal spike initiation can be independent of direct synaptic inputs, but instead be due to local integration of environmental signals and activity-dependent changes in membrane excitability (Pinault, 1995; Bucher and Goaillard, 2011; Bucher, 2015). This is often associated with modulatory effects mediated by high-affinity non-synaptic receptors, either G protein-coupled or ionotropic. For example, in hippocampal and cortical neurons, distal spike initiation is thought to arise from spillover-activation of high-affinity GABA_*A*_ receptors, sometimes in conjunction with axonal gap junction coupling and persistent Na^+^ currents (Avoli et al., 1998; Keros and Hablitz, 2005; Bahner et al., 2011; Bukalo et al., 2013; Muller et al., 2018). In the crustacean stomatogastric nervous system (STNS), distal spike initiation in descending, sensory, and motor axons occurs in response to aminergic or peptidergic modulation, and affects sensorimotor integration (Daur et al., 2009; Stadele and Stein, 2016; Stadele et al., 2018), circuit activity (Bucher et al., 2003; Goaillard et al., 2004; Daur et al., 2009), and motor output to muscles (Meyrand et al., 1992; Bucher et al., 2003; Ballo and Bucher, 2009).

Different spike initiation sites in the same neuron can be functionally separated and operate independently (Calabrese and Kennedy, 1974; Dugladze et al., 2012), but in many cases, they influence each other. In some neurons, distal spiking is elicited in response to repetitive activity propagated from proximal sites (Meyrand et al., 1992; Le et al., 2006; Sheffield et al., 2011, 2013; Suzuki et al., 2014; Elgueta et al., 2015). In others, spikes propagated from one site suppress initiation at the other (Calabrese, 1980; Nagy et al., 1981; Maranto and Calabrese, 1983; Pinault, 1995; Cattaert and Bevengut, 2002; Weidner et al., 2003; Blitz and Nusbaum, 2008). Neither the degree to which such interactions shape neuronal output activity nor the underlying cellular mechanisms are well understood. We address these aspects in the pyloric dilator (PD) neuron in the STNS. We show that output activity of the pyloric dilator (PD) motor neuron in the STNS is shaped by suppressive interactions between centrally generated bursting activity and dopamine (DA)-elicited spike initiation in the peripheral axon. As the two initiation sites are electrotonically well separated, the bidirectional interactions depend on propagating spikes, and are mediated by different mechanisms. Some of these results were previously published in abstract form (Daur et al., 2015).

## Materials and Methods

### Experimental Preparation

All experiments were performed on the STNS of adult (∼500 g) lobsters, *Homarus americanus*, of either sex. Animals were obtained from Yankee Lobster Co. in Boston, MA, United States, or from local seafood stores in Newark, NJ, United States, and kept unfed in tanks at 10–13°C. Prior to dissection, animals were cold-anesthetized in ice for ∼15 min. The STNS was dissected from the stomach and pinned in a transparent Sylgard-lined (Dow Corning) 100 mm experimental dish in physiological saline. Saline composition was as follows (in mM): 479.12 NaCl, 12.74 KCl, 13.67 CaCl_2_, 10 MgSO_4_, 3.91 Na_2_SO_4_, and 10 HEPES. The pH was adjusted to 7.4 –7.5.

**FIGURE 1:**
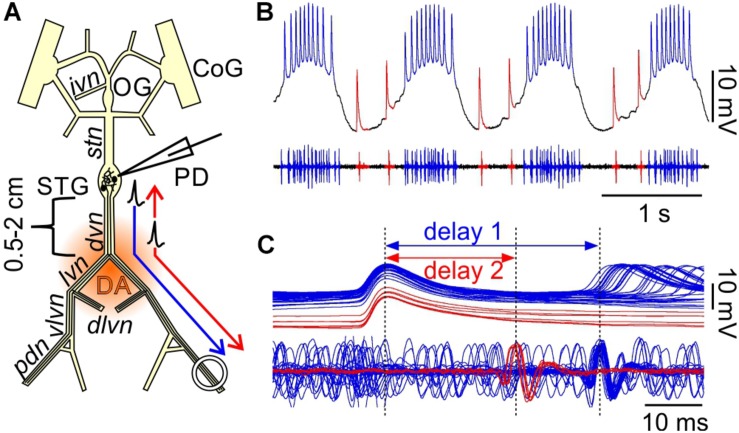
Orthodromic and antidromic propagation of spikes in the PD neurons. **(A)** Schematic of the isolated STNS as used in the *in vitro* experiments described here. In most experiments, the PD neurons were recorded intracellularly from the soma in the STG, and extracellularly from a petroleum jelly well around the *pdn*, at 4–5 cm distance. Bursts of spikes are generated in the STG and propagate orthodromically to the distal recording site (blue arrow). In addition, DA can elicit spikes throughout most of the axons in the peripheral nerves, but the dominant site of peripheral spike initiation is in the distal *dvn*. Peripherally elicited spikes propagate both antidromically to the STG and orthodromically to the distal targets (red arrows). CoG, commissural ganglia; OG, esophageal ganglion; STG, stomatogastric ganglion; PD, pyloric dilator neurons; *ivn*, inferior ventricular nerve; *stn*, stomatogastric nerve; *dvn*, dorsal ventricular nerve; *lvn*, lateral ventricular nerve; *dlvn*, dorsal lateral ventricular nerve; *vlvn*, ventral lateral ventricular nerve; *pdn*, pyloric dilator nerve. **(B)** Simultaneous intracellular recording from the PD soma and extracellular recording from the *pdn*. Centrally generated spikes (blue) occur during bursts on top of a rhythmic slow wave depolarization. Peripherally generated spikes (red) occur only during the interval between bursts. **(C)** Overlaid sweeps of the same recording shown in **(B)**, triggered from the peak of each spike in the soma recording. There are distinct delays for the two types of spikes. Centrally generated spikes show a longer delay (1) between soma and *pdn* than peripherally generated ones (2).

A schematic of the STNS with the main nerves is shown in Figure 1A, with the established nomenclature (Maynard and Dando, 1974). The esophageal ganglion (OG), the paired commissural ganglia (CoG), and the proximal inferior ventricular nerve (*ivn*) contain neuromodulatory neurons with axons that project to the STG. All experiments involved the PD neurons, which have their cell bodies in the STG. The PD neurons have a dual role as part of the pacemaker kernel of the pyloric central pattern generator and as motor neurons innervating pyloric dilator muscles (Marder and Bucher, 2007). In some experiments, we also recorded from the anterior burster (AB) neuron. There are two copies of PD, and they are not bilateral homologs but each projects to muscles on both sides of the stomach. Therefore, all nerves in the path to PD innervated muscles contain both axons. We discarded all muscles but kept the axon path to the pyloric dilator nerve (*pdn*) intact. In animals of the size used, the path length from the STG to the *pdn* is ∼4–5 cm, with a spike conduction delay of ∼30–50 ms (Bucher et al., 2003, 2005; Ballo and Bucher, 2009; Ballo et al., 2012).

### Electrophysiological Recordings

In most experiments, a PD neuron was recorded intracellularly from the soma in the STG (sometimes simultaneously with the AB neuron), and extracellularly from the *pdn*. The STG was desheathed with the tip of a fine tungsten wire. PD neurons were identified by their characteristic waveform and correspondence of spiking activity with the extracellular nerve recordings. AB neurons were identified by their characteristic waveforms, soma size, and burst phasing in relation to PD. Only those recordings of PD and AB were analyzed in which the membrane potential troughs during bursting activity were more negative than −50 mV, and the slow wave depolarizations at least 10 mV in amplitude.

In some experiments, we obtained intracellular recordings from the PD axon in the *dvn*, at 0.5–2 cm distance to the STG. To facilitate access to the axons, the nerve was mechanically desheathed and slit with a tungsten wire as described before (Ballo and Bucher, 2009). As in soma recordings, PD neurons were identified by their characteristic spike pattern and correspondence of spiking with the extracellular nerve recordings. Only those recordings were analyzed in which the membrane potential troughs during bursting activity were more negative than −55 mV, and the spikes were overshooting, indicating no impalement damage.

During all recordings, preparations were superfused with saline cooled to 12°C by a custom-made Peltier cooling device. For intracellular soma recordings, sharp glass electrodes were pulled with a Flaming-Brown P-97 puller (Sutter Instruments) and filled with 0.6 M K_2_SO_4_ and 20 mM KCl to minimize alteration of chloride conductances present in the STG. These electrodes yielded tip resistances of 20–30 MΩ. For intracellular recordings of the PD axon, electrodes with sharper tips were used, filled with 3 M KCl. These electrode yielded tip resistances of 20–30 MΩ. Signals were amplified using Axoclamp 2B and 900A amplifiers (Molecular Devices).

Extracellular recordings from the *pdn* were obtained by placing stainless steel wires inside and outside of a petroleum jelly well around the distal part of the nerve, and amplifying the signals with a differential AC amplifier (A-M Systems, model 1700). Electrical nerve stimulation of the *pdn* or *dvn* was achieved through the same type of electrodes, using an isolated pulse stimulator (A-M Systems, model 2100). Pulse durations were between 200 and 500 μs and the amplitude was adjusted to be just enough above threshold to sustain repetitive stimulation.

All electrophysiological signals were acquired using a micro 1401 digitizing board (Cambridge Electronic Design) and the accompanying Spike2 software (versions 6-8). Stimulation protocols were generated using either the time settings on the stimulator, or the sequencer interface of the digital-to-analog converter of the micro 1401, connected to the trigger input of the stimulator.

### Drug Applications

In some experiments, descending modulatory input to the STG or activity in the STG itself was blocked with 1 μM tetrodotoxin citrate (TTX; Biotium) and 750 mM sucrose (Sigma) in a petroleum jelly well around the stomatogastric nerve (*stn*) or the STG. For bath application of 1 μM DA (dopamine; 3-hydroxytyramine hydrochloride; Sigma), freshly made stock solutions were diluted in saline right before use. Application was done through the superfusion system with the use of switching manifolds.

### Data Analysis

Primary data analysis to extract spike times, spike and burst frequencies, and voltage trajectory measures was performed using Spike2 and programs written in its script language. Secondary analyses, statistical tests, and plots were generated in SigmaPlot (version 12.0, Systat Software). Statistical tests used were One Way (1W) or Two Way (2W) Repeated Measures Analysis of Variance (RM-ANOVA), with subsequent Holm–Sidak *post hoc* comparisons, or Friedman Repeated Measures ANOVA on Ranks (RM-Rank-ANOVA), with subsequent Tukey *post hoc* comparisons. Significance was assumed at *p* < 0.05, and is indicated as ^∗^ (*p* < 0.05), ^∗∗^ (*p* < 0.01), and ^∗∗∗^ (*p* < 0.001). Error bars indicate Standard Error of Means. Figures were produced in CorelDRAW (versions X7 and X8, Corel) and Canvas (version 11, ACD Systems).

### Multicompartment Modeling of the PD Axon and Pyloric Pacemaker

This study includes two sets of computational models. The first is a circuit model of the pyloric pacemaker group AB and two PD neurons, in which we examined the effect of ectopic spiking in the PD axon on centrally generated bursting activity in the pacemaker group. The second is a model of a single PD axon, in which we examined the effect of burst-pattern stimulations of the axon on the ectopic tonic spiking activity produced by DA neuromodulation. All simulations were done in NEURON^1^ + Python (version 7.6.7; Python Software Foundation, version 3.71) (Hines et al., 2009).

#### The Pyloric Pacemaker Model

The circuit model of the pyloric pacemaker group included three electrically coupled neurons and was modified from the AB and PD model neurons in Soto-Treviño et al. (2005), but with a simpler, more generic set of ionic conductances (Table 1). The AB neuron was modeled as a single compartment, representing the soma and neurite (S/N), and included a leak current (*I*_*Leak*_), a slow and inactivating Ca^2+^ current (*I*_*CaS*_), a slow non-inactivating K^+^ current (*I*_*KS*_), and the modulator-activated inward current (*I*_*MI*_). The PD neurons each had 4 compartments: one S/N compartment, connected to a 3-compartment axon. The proximal axon compartment was modeled with twice the membrane surface area each of the two distal compartments. This provided enough electrotonic separation for S/N subthreshold voltage fluctuation not to interfere with distal spike initiation, and stimulus artifacts from the distal compartment not to be transmitted to the S/N compartment. The S/N compartment included the same currents as AB, plus the hyperpolarization-activated inward current (*I*_*h*_), and the axial current from the proximal axon compartment (*I*_*axial*_). To introduce a small amount of heterogeneity, the S/N compartments of the two PD neurons differed in their *I*_*Leak*_ reversal potential by 1 mV. The axon compartments included *I*_*Leak*_, an instantaneous Na^+^ current (*I*_*Na*_), a delayed rectifier K^+^ current (*I*_*Kd*_), and *I*_*axial*_ from adjacent compartments. The three neurons were symmetrically electrically coupled through their S/N compartments (*I*_*elec*_). In isolation, AB produced slow wave oscillations, whereas PD only produced tonic spiking. To study the effect of antidromic activity, 1 ms current pulses of 1.5 nA amplitude were injected either simultaneously into the distal axon compartments of both PDs to elicit spikes, or directly into AB to mimic the effect of antidromic spikes on its membrane potential. All ionic currents were based on the standard Hodgkin–Huxley formalism, with activation (and inactivation) state variables (x) obeying the standard equation

**TABLE 1:** Pacemaker circuit model compartments and currents.

**Cell compartment**	**Ionic current**	***ḡ*_*x*_ [nS]**	***E*_*x*_ [mV]**
*AB (S/N) C_*m*_* = 60 nF	*I*_*Leak*_	12	−58
	*I*_*MI*_	12 (8.4 – 12)	−10
	*I*_*CaS*_	4	120
	*I*_*KS*_	45	−80
*PD (S/N) C_*m*_* = 40 nF	*I*_*Leak*_	20	−58 (PD_1_)/ −59 (PD_2_)
	*I*_*MI*_	6.7 (4.7 – 6.7 = $\frac{5}{9}\,{\bar{g}}_{M\,I\,\_\,A\,B}$)	−10
	*I*_*CaS*_	4	120
	*I*_*KS*_	20	−80
	*I*_*h*_	3	−10
	*I*_*axial*_	8	N/A
*PD (Axon) C_*m*_* = 10 nF (axon1)/ 5 nF (axon2,3)	*I*_*Leak*_	5 (axon1)/ 2.5 (axon2,3)	−65 (axon1)/ −67 (axon2,3)
	*I*_*Na*_	1500 (axon1)/ 750 (axon2,3)	50
	*I*_*Kd*_	300 (axon1)/ 150 (axon2,3)	−80
	*I*_*axial*_	8 (axon1)/ 1 (axon2,3)	N/A
*Electrical coupling (AB-PD; PD-PD)*	*I*_*elec*_	6	N/A

$$\frac{d\,x}{d\,t}=\frac{x_{\infty }\,\left(V\right)-x}{\tau_{x}\,\left(V\right)}.$$

Maximal conductances and reversal potentials for all currents are listed in Table 1. The equations for activation and inactivation state variables (*x*_8_ and *τ*_*x*_) are provided in Table 2.

**TABLE 2:** Gating parameters of ionic currents in the pacemaker circuit model.

**Ionic Current**	***x***	***x*_∞_**	***τ_*x*_* [ms]**
*I*_*Na*_	*m*^3^	$\frac{1}{1+e\,x\,p\,\left(-0.08\,\left(x+18\right)\right)}$	0
	*h*	$\frac{1}{1+e\,x\,p\,\left(0.13\,\left(x+28\right)\right)}$	2
*I*_*Kd*_	*m*^4^	$\frac{1}{1+e\,x\,p\,\left(-0.2\,\left(x+23\right)\right)}$	$2+\frac{14}{1+e\,x\,p\,\left(-0.2\,\left(x+23\right)\right)}$
*I*_*CaS*_	*m*^3^	$\frac{1}{1+e\,x\,p\,\left(-\frac{x+54.5}{3}\right)}$	$12+\frac{12}{1+e\,x\,p\,\left(-\frac{x+54.5}{3}\right)}$
	*h*	$\frac{1}{1+e\,x\,p\,\left(\frac{x+51}{2}\right)}$	$500+\frac{2000}{1+e\,x\,p\,\left(\frac{x+51}{2}\right)}$
*I*_*MI_AB*_	*m*	$\frac{1}{1+e\,x\,p\,\left(-\frac{x+55}{5}\right)}$	20
*I*_*MI_PD*_	*m*	$\frac{1}{1+e\,x\,p\,\left(-\frac{x+55}{2}\right)}$	5
*I*_*KS*_	*m*^2^	$\frac{1}{1+e\,x\,p\,\left(-\frac{x+56}{2}\right)}$	$2000-\frac{1500}{1+e\,x\,p\,\left(-\frac{x+56}{2}\right)}$
*I*_*h*_	*m*^2^	$\frac{1}{1+e\,x\,p\,\left(\frac{x+70}{5}\right)}$	$700-\frac{250}{1+e\,x\,p\,\left(\frac{x+70}{5}\right)}$

### The PD Axon Model

We previously published a model of the PD axon that reproduced DA effects on spike conduction (Zhang et al., 2017). This model contained *I*_*Leak*_, *I*_*Na*_, *I*_*Kd*_, *I*_*h*_, a fast transient K^+^ current (*I*_*A*_), and the Na^+^/K^+^ pump current. Here, we used the same model, with two modifications. First, the effect of DA on the axon was simulated by increasing the maximal conductance of *I*_*h*_ to 0.25 mS/cm^2^ [rather than the 0.1 mS/cm2 used in Zhang et al. (2017)]. This increased level reproduced the rate of tonic spiking activity produced by 1 μM DA in the biological PD axon (Bucher et al., 2003). Second, the length of the axon was limited to 1 mm, divided into 11 compartments (rather than 1 cm divided into 101 compartments, as in Zhang et al., 2017). This second modification was simply to reduce computation time, since the length of the axon was irrelevant for the results obtained. Burst stimulation patterns were produced at one end of the axon with 2 ms current pulses of 5 nA amplitude, and recordings were made from the midpoint of the axon.

## Results

### Centrally and Peripherally Generated Spikes Are Readily Identified by Differences in Conduction Delay

In *H. americanus*, DA elicits peripheral spike initiation in the PD motor axons (Bucher et al., 2003; Ballo and Bucher, 2009; Ballo et al., 2010). In the presence of centrally generated rhythmic bursting activity, these spikes occur exclusively during the interval between bursts. While focal DA application can elicit spikes along almost the entire length of the PD axons, spikes are consistently generated close to the *dvn/lvn* junction (0.5–2 cm from the STG, Figure 1A) during bath applications (Bucher et al., 2003).

Simultaneous intracellular PD soma and extracellular *pdn* recordings during mixed activity allowed a clear distinction between centrally generated bursting and “extraburst” peripheral spiking (Figure 1B). Note that STG neurons are typical unipolar invertebrate neurons with unexcitable somata, and therefore only show substantially attenuated spikes, usually on top of slow wave depolarizations which, in contrast, are only minimally attenuated (Golowasch and Marder, 1992). The different sites of spike initiation can be confirmed by determining the conduction delay between soma and nerve recording (Bucher et al., 2003; Figure 1C). Spikes generated during bursts on top of slow wave depolarizations show a longer delay, indicating that they are generated in the STG and propagate the entire length of the nerves to the *pdn*. Extraburst spikes show a shorter delay, indicating that they are generated in the axon at some distance to the STG, and then propagate both antidromically to the soma, and orthodromically to the *pdn* (arrows in Figure 1A). The positive delay indicates that the peripheral spike initiation site is closer to the soma than to the *pdn*, as no delay would indicate equidistance, and negative delay would indicate a more distal initiation site.

### Antidromic Spikes Interfere With Slow Central Burst Generation

In *H. americanus*, the isolated STNS produces continuous pyloric rhythms with cycle frequencies of ∼0.4–1 Hz (Bucher et al., 2005, 2006). This activity is characterized by bursts in the pacemaker neurons (AB and PD), followed by bursts in follower neurons which rebound from inhibition by the pacemaker, and is dependent on descending neuromodulatory input to the STG (Marder and Bucher, 2007). When descending input is blocked, follower neurons fall silent, but AB and PD continue to cycle at a lower frequency (∼0.1–0.4 Hz) (Thirumalai and Marder, 2002; Bucher et al., 2003). These two states of the pyloric circuit *in vitro* are somewhat representative of fast and slow activity in the intact animal, but with some caveats. Pyloric activity in the intact animal is also dependent on hormonal modulation via the hemolymph (Marder and Bucher, 2007), which is absent *in vitro*. Inhibitory neuromodulators can substantially slow or disrupt rhythmic pyloric activity (Claiborne and Selverston, 1984; Cazalets et al., 1987; Skiebe and Schneider, 1994; Pulver et al., 2003; Kwiatkowski et al., 2013), but that is likely not associated with a complete absence of excitatory neuromodulation, as is the case *in vitro*, when all inputs to the STG are blocked. Nevertheless, we used these states as approximations of different modulatory conditions in which the pyloric rhythm is either fast or slow.

**FIGURE 2:**
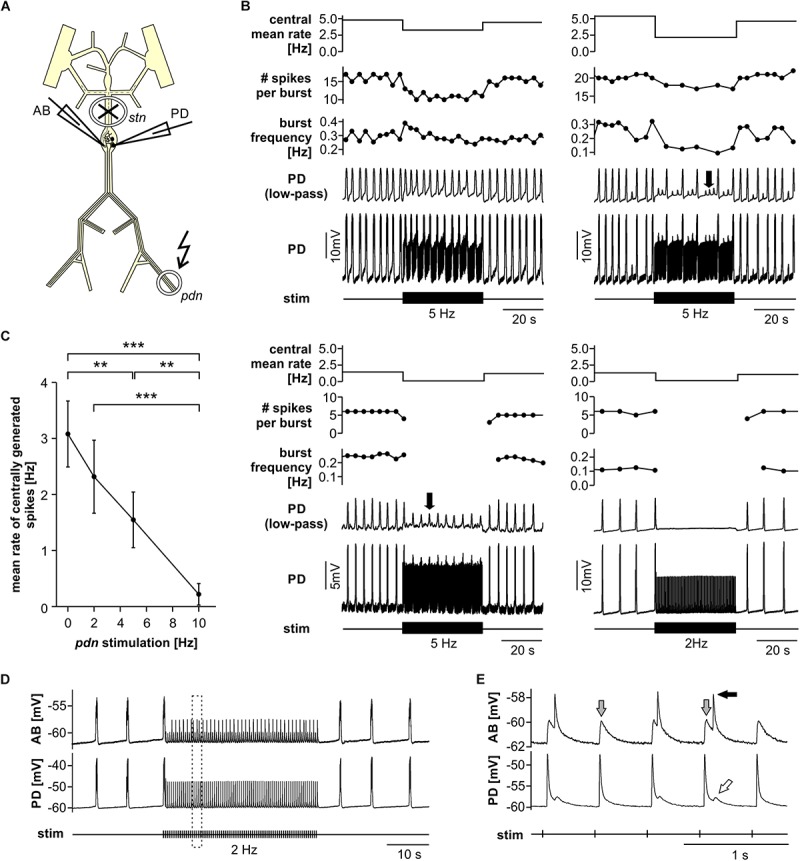
Suppression of slow rhythmic activity of the pyloric pacemaker group by antidromic PD axon stimulation. **(A)** Schematic of the STNS. Descending modulatory input through the *stn* was blocked and antidromic spikes were elicited by stimulating the *pdn* (kinked arrow). **(B)** Four examples of intracellular recordings of PD during antidromic stimulation. Low-pass filtered PD voltage traces were generated to visualize slow-wave oscillations by plotting the moving average of voltage (time constant: 100 ms), and are not shown at the same scale as the original recordings. Arrows point to small slow wave oscillations that did not give rise to bursts of spikes. Burst frequency, number of spikes per burst, and central mean rate were calculated after removing stimulated antidromic spikes from the original spike detection. Burst frequency plots are instantaneous frequencies obtained as the inverse of intervals between the first spike of a burst and the first spike in the preceding burst. Central mean rates were obtained from 40 s windows before, during, and after stimulation, and are shown as skyline plots. **(C)** Central mean rate (mean rate of centrally generated spikes) decreased with increasing antidromic stimulation frequency (*n* = 8; 1W-RM-ANOVA, *p* < 0.001). Asterisks show significance obtained from *post hoc* pairwise comparisons. **(D)** Example simultaneous AB and PD recording during antidromic stimulation, showing complete disruption of centrally generated bursts. The dashed box indicates the time range shown in **(E)**. **(E)** Expanded section of the recording shown in **(D)**. Antidromic PD spikes caused spikelets in the electrically coupled AB neuron (gray arrows), that could elicit spikes in AB (black arrow), which in turn caused spikelets in PD (white arrow).

When modulatory inputs are blocked and DA is bath applied onto the entire STNS, pacemaker burst frequency initially increases. However, with some delay, peripheral spike initiation increases and burst generation decreases and eventually ceases completely (Bucher et al., 2003). Therefore, we asked if antidromic propagation of peripherally generated spikes directly causes inhibition of central burst generation, even when DA is not present in the STG. We blocked descending inputs to the STG and electrically stimulated the distal *pdn* to elicit antidromic spikes, while recording intracellularly from a PD soma (Figure 2A). Under these conditions, even relatively low frequency antidromic spiking had an inhibitory effect on centrally generated bursting in PD. Figure 2B shows four different ways bursting was disrupted. Stimulus artifacts were minimal in all intracellular recordings, and antidromic spikes occurred with a consistent delay from stimulation (30–50 ms, dependent on preparation). We excluded those spikes from the calculation of burst frequency, number of spikes per burst, and the mean rate of centrally generated spikes. Slow wave oscillations were obtained by low-pass filtering. In all experiments and at all frequencies, antidromic spikes had stable amplitudes throughout the 40 s stimulation interval, suggesting that membrane refractoriness and the ability to generate spikes were not affected. In some cases, there was little effect on burst frequency, but the number of spikes per burst decreased during antidromic stimulation, associated with a decrease in slow wave amplitude (Figure 2B, upper left). In others, the predominant effect was a decrease in burst frequency (Figure 2B, upper right). However, in some of these cases, smaller slow wave oscillation that did not give rise to spikes were still present in the prolonged intervals between bursts (arrow in Figure 2B, upper right). Cases in which central spike initiation was completely suppressed either still showed diminished slow wave oscillations (Figure 2B, lower left, arrow), or not (Figure 2B, lower right). There was variability, both across experiments and across stimulation frequencies within experiments, of whether the effect on burst frequency or the effect on spikes per burst was dominant. Therefore, we used the mean rate of centrally generated spikes (that is independent of the precise temporal structure) as a measure of activity (top plots in Figure 2B). Figure 2C shows that this rate declined linearly with increasing stimulation frequency. The linear relationship suggests that, on average, stimulation above 10 Hz should reliably completely disrupt central burst and spike initiation.

The single interneuron AB is the dominant oscillator in the pacemaker group, and both PD neurons are strongly electrically coupled to AB and to each other (Marder and Bucher, 2007; Daur et al., 2016). The reduced amplitude slow wave oscillations in PD, shown in Figure 2B, could either be due to reduced oscillations in AB, or to a decreased responsiveness of PD to stable AB oscillations. In three experiments, we therefore recorded simultaneously from AB and PD. In all three experiments, antidromic PD stimulation reduced or disrupted AB slow wave oscillations (Figure 2D), and we consistently observed AB responses corresponding to individual antidromic PD spikes (Figure 2E). These responses were spikelets (gray arrows in Figure 2E), and were sometimes sufficient to cause the AB neuron to fire after a small delay (black arrow in Figure 2E). Spikes generated in AB in turn then caused spikelets in the PD recordings (white arrow in Figure 2E). These interactions are an indication that fast signals are efficiently transmitted between PD and AB, and that antidromic PD spikes can have a fairly global influence on AB.

### Fast Rhythmic Pyloric Activity Is Robust to Antidromic Spiking

**FIGURE 3:**
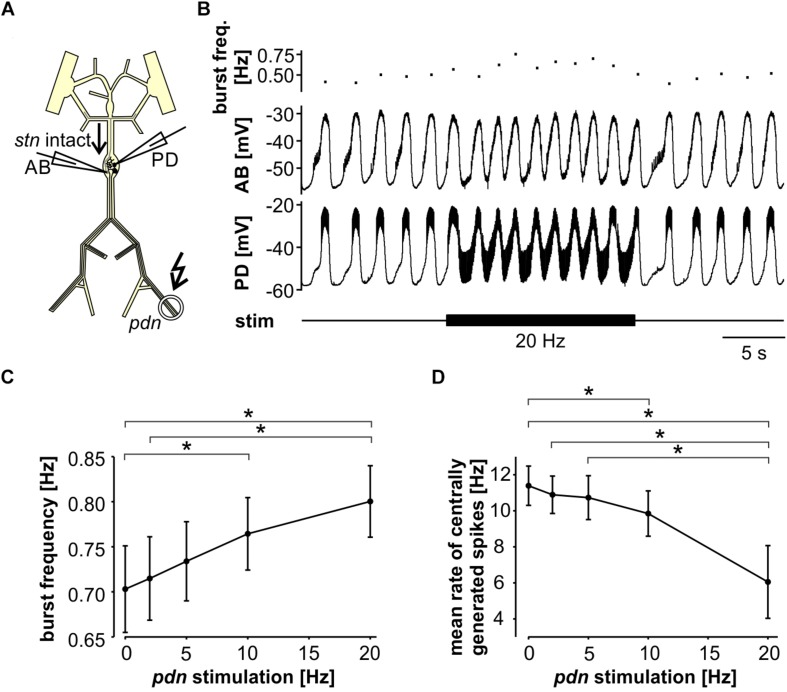
Robustness of fast pyloric rhythmic activity to antidromic PD axon stimulation. **(A)** Schematic of the STNS. Descending modulatory input through the *stn* was left intact and antidromic spikes were elicited by stimulating the *pdn* (kinked arrow). **(B)** Intracellular AB and PD recording, showing a slight increase in burst frequency during antidromic PD stimulation. **(C)** Burst frequency increased with increasing antidromic stimulation frequency (*n* = 8; RM-Rank-ANOVA, *p* < 0.001). Asterisks show significance obtained from *post hoc* pairwise comparisons. **(D)** Mean rate of centrally generated spikes decreased with increasing antidromic stimulation frequency (*n* = 8; RM-Rank-ANOVA, *p* < 0.001). Asterisks show significance obtained from *post hoc* pairwise comparisons.

Next, we tested the influence of antidromic spiking on fast pyloric activity with intact descending inputs (Figure 3A). Intracellular soma recordings of PD alone, or simultaneous recordings of PD and AB, showed effects of antidromic spikes on slow wave oscillation amplitudes and burst frequency when stimulated at relatively high frequency (Figure 3B). In contrast to the effects seen when descending modulatory inputs were blocked, bursting activity never ceased when inputs were intact. In fact, with intact inputs, burst frequency was slightly but significantly increased from the unstimulated pattern when antidromic spikes were delivered at 10 or 20 Hz (Figure 3C). We also analyzed the effect on mean spike rates (Figure 3D). Particularly at higher stimulation frequencies, antidromic spikes replaced some spikes generated centrally at the peak of the slow wave oscillations. At 10 and 20 Hz stimulation, the mean centrally generated spike rate was significantly lower than the unstimulated pattern, despite the increase in burst frequency.

### A Pyloric Pacemaker Model Replicates the Effects of Antidromic Spiking on Rhythm Generation

**FIGURE 4:**
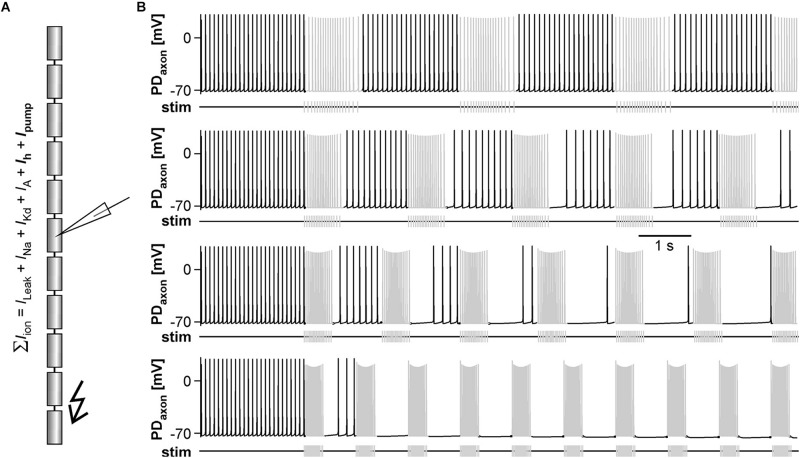
The effects of antidromic spikes on fast pyloric activity in the model pacemaker circuit. **(A)** Schematic of the multi-compartment circuit model and the rhythmic activity it produced. Resistor symbols indicate electrical coupling between the soma-neurite (S/N) compartments. Recording sites are indicated with electrode symbols, stimulation sites with kinked arrows. **(B)** Voltage responses to PD axon or AB stimulations during the interval between bursts. Depolarizing current injection of 1 ms amplitude into the distal axon elicited antidromic spikes that were attenuated to non-overshooting but substantial depolarizations in the PD_*S/N*_ compartment, and transmitted to AB as much smaller depolarizations through electrical coupling (upper traces). Direct injection into AB caused small depolarizations that were transmitted to PD through electrical coupling with little attenuation (lower traces). **(C)** AB voltage traces and instantaneous burst frequencies during antidromic PD axon stimulation at different frequencies. **(D)** AB voltage traces and instantaneous burst frequencies during direct AB stimulation at different frequencies. **(E)** Mean burst frequencies at different frequencies of PD axon and AB stimulation. **(F)** Mean rate of centrally generated spikes at different frequencies of PD axon and AB stimulation. Stimulated antidromic spikes were excluded.

To address whether the circuit structure of the pyloric pacemaker kernel and common ionic mechanisms are sufficient to give rise to the influence of ectopic spikes on centrally generated activity, we used a simplified circuit model of the AB and two PD neurons (Figure 4A). The AB neuron was represented with a single soma-neurite compartment as a non-spiking oscillator, whereas each PD neuron in addition was connected to a 3-compartment axon that produced spikes. As in the biological circuit, the three neurons were electrically coupled. The model produced activity with similar temporal features as the pyloric pacemaker, and we modeled different modulatory states that produced fast or slow activity by varying the modulator-activated inward current, *I*_*MI*_. *I*_*MI*_ is the primary activator of neuropeptide-induced rhythmic activity in the STG (Sharp et al., 1993a, b; Swensen and Marder, 2000; Marder and Bucher, 2007; Daur et al., 2016), and increases pacemaker frequency (Sharp et al., 1993b).

We started with a level of *ḡ*_*MI*_ that gave rise to regular rhythmic activity with a cycle frequency of 0.71 Hz in the unperturbed state, similar to fast pyloric activity in the biological circuit. Ectopic spikes were then produced by stimulating the distal PD axon compartments. As in the biological neurons, antidromic spikes were attenuated in the soma and evoked spikelets of a few millivolt amplitude in the AB neuron (Figure 4B, upper traces). In order to test directly if brief depolarizations of AB were sufficient to influence pacemaker oscillations and bursting in PD, we also generated brief current injections directly into the AB neuron. These injections evoked depolarizing potentials of a few millivolts amplitude and a similar time course as electrically transmitted spikelets, and in turn evoked small potentials in the PD neurons, but no active responses (Figure 4B, lower traces). We stimulated either the PD axon (Figure 4C) or the AB neuron (Figure 4D) at different frequencies and measured the effect on burst frequency and mean rate of centrally generated spikes (Figures 4E,F). As in the experimental data (Figure 3), burst generation was fairly robust to stimulation. Burst frequency increased linearly, but maximally by a few percent. However, the rate of centrally generated PD spiking decreased linearly. Both effects were larger with PD axon stimulation than with AB stimulation, potentially because of asymmetric coupling between AB and PD due to impedance mismatch (see *ḡ*_*Leak*_ values in Table 1).

**FIGURE 5:**
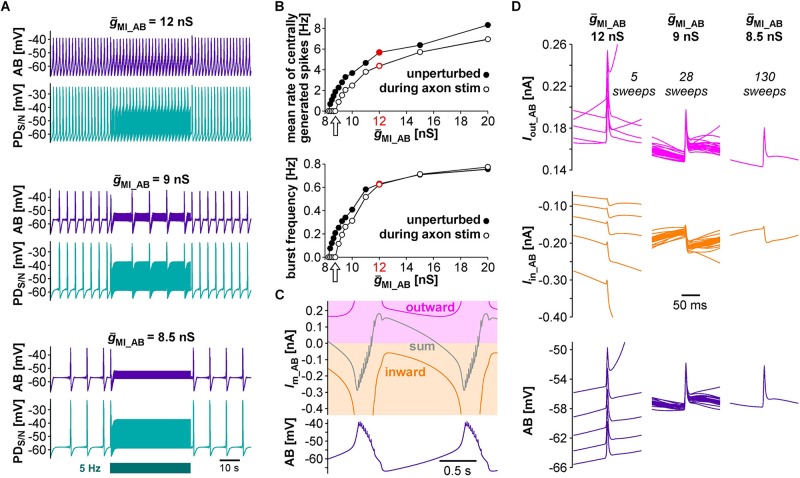
The effects of antidromic spikes on model pacemaker activity at different burst frequencies. **(A)** 5 Hz PD axon stimulation in the normal circuit model (upper traces), and after reduction of *ḡ*_*MI*_ in both AB and PD (lower traces; *ḡ*_*MI_PD*_ was kept at 5/9 of *ḡ*_*MI_AB*_). **(B)** The effect of *ḡ*_*MI*_ on mean rate of centrally generated spikes and burst frequency in the unperturbed circuit, and during 5 Hz axon stimulation. Values at the level of *ḡ*_*MI*_ used for Figure 4 are shown in red. **(C)** Voltage and membrane current (*I*_*m*_) trajectories in AB (*ḡ*_*MI*_ = 12 nS). Inward current (orange) is the sum of *I*_*CaS*_, *I*_*h*_, and *I*_*MI*_, outward current (magenta) is *I*_*KS*_. The sum of inward and outward currents is shown in gray. **(D)** Overlaid traces of AB voltage and current trajectories from each interspike interval during the latter two thirds of a burst cycle during antidromic PD stimulation. Different columns are from different *ḡ*_*MI*_ levels, with the number of overlaid traces indicated.

Next, we tested the effect of antidromic spikes on different burst frequencies. To mimic modulatory states that produce slower or faster pyloric frequencies, we reduced or increased the conductance level of *I*_*MI*_ proportionally in both AB and PD. At lower *ḡ*_*MI*_ values and burst frequencies, antidromic spiking at a fixed frequency of 5 Hz disrupted or greatly reduced central bursting (Figure 5A). Like in the experimental data, both the burst frequency and the number of spikes per burst varied across conditions. Therefore, we also determined the mean rate of centrally generated spikes as a measure of circuit activity. Figure 5B shows the mean rates and burst frequencies as a function of *ḡ*_*MI*_ values for both unperturbed activity and during axon stimulation. The mean rate was always lower during axon stimulation, even at increased *ḡ*_*MI*_ values at which burst frequency was greater during stimulation than in the unperturbed state. This was due to a reduction in the number of spikes per burst, and it replicated the experimental findings for fast pyloric activity (Figures 3C,D). When *ḡ*_*MI*_ was reduced by more than 25% compared to the original value, centrally generated activity ceased completely during axon stimulation (arrows in Figure 5B).

In general, changes in membrane potential occur when the total inward and outward currents are not at balance. In the case of slow oscillations in the pyloric pacemaker model, small but continuous changes in inward and outward currents following a burst slowly moved the total current to more negative values until the next burst occurred (Figure 5C). Therefore, the effects of antidromic spikes on bursting mean that very brief but repetitive voltage events interfered with much slower current trajectories. Figure 5D shows AB voltage and current trajectories during antidromic PD stimulation for the same *ḡ*_*MI*_ values as in Figure 5A. Overlaid traces are from each interspike interval during the latter two thirds of a burst cycle. During fast bursting (large *ḡ*_*MI*_), consecutive sweeps showed large offsets because antidromic spike-evoked deflections simply rode on top of largely unperturbed slower changes. At intermediate burst frequency, when antidromic stimulation reduced but did not eliminate centrally generated activity, offset was reduced but still present. With *ḡ*_*MI*_ values at which antidromic stimulation eliminated bursting, slow dynamics was completely eliminated, resulting in virtually identical voltage and current trajectories in each interspike interval.

### Centrally Generated Bursts Inhibit Peripheral Spike Initiation

**FIGURE 6:**
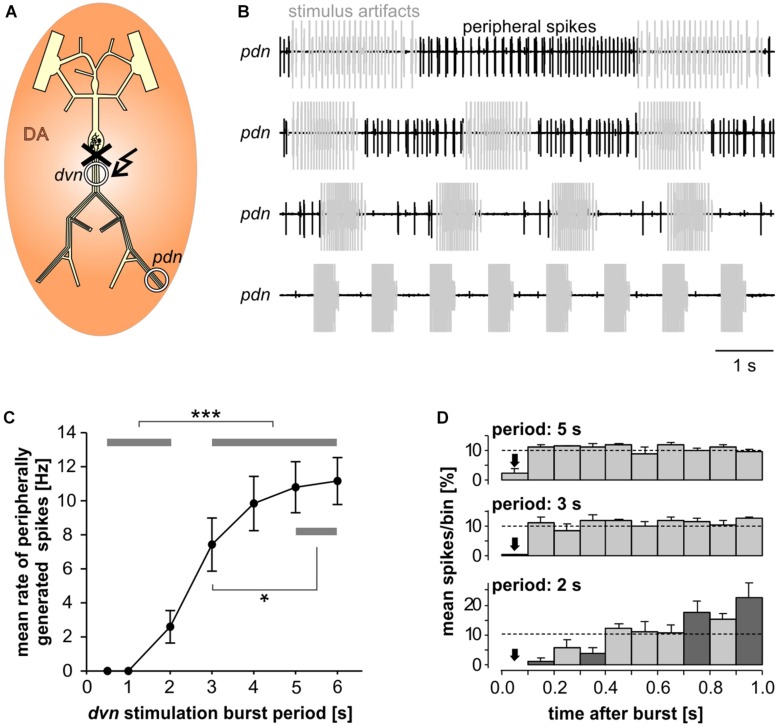
Inhibition of peripheral spiking by centrally generated bursts. **(A)** Schematic of the STNS. The peripheral nerves were disconnected from centrally generated activity by cutting the *dvn* close to the STG. Bursting activity was then elicited by stimulation of the cut end of the *dvn* (kinked arrow), while DA was bath applied and PD activity recorded extracellularly from the *pdn*. **(B)** Extracellular recordings during burst stimulations (gray) at different cycle periods. Peripheral spike initiation increased with increasing periods. **(C)** Stimulation period had a significant effect on the mean rate of peripherally generated spikes (*n* = 10; 1W-RM-ANOVA, *p <* 0.001). *Post hoc* testing revealed significant differences for all pairwise comparisons of any of the periods between 0.5, and 2 s with any of the periods between 3 and 6 s (*p <* 0.001, asterisks), and for pairwise comparisons between 3 s period with any of the periods between 4 and 6 s (p *<* 0.05, asterisk). **(D)** Mean normalized spike counts during the first second after a burst, divided into ten 100 ms bins, for 2, 3, and 5 s burst stimulation periods. Analysis included only experiments in which peripheral spiking occurred at all three periods (*n* = 7). In each experiment, the number of spikes per bin was normalized to the total number of spikes in the 1 s window. Dashed lines at 10% indicate the expected level if peripheral spiking had been equal throughout the analysis window. 2W-RM-ANOVA revealed differences across bins and a significant interaction between bins and stimulation periods (*p <* 0.001 for both). At 2 s stimulation period, *post hoc* testing showed significant differences in 21 of the 45 pairwise comparisons across bins, indicating that peripheral spiking increased over the course of the whole 1 s analysis window. At 3 and 5 s stimulation periods, pairwise comparisons only showed differences between the first bin and a subset of the other bins, indicating that a time-variant effect on peripheral spiking did not exceed 100 ms. *Post hoc* testing also showed that none of the bins differed between 3 s and 5 s periods. However, some bins (darker shading) were different between 2 s and both 3 s and 5 s periods, indicating that the temporal structure of peripheral spiking was different at 2 s, compared to 3 s and 5 s.

In the absence of centrally generated activity, lower than nanomolar concentrations of DA can elicit peripheral spikes. However, during robust and fast rhythmic pyloric activity, peripheral spiking is much less prevalent, even at micromolar concentrations of DA (Bucher et al., 2003). We therefore quantified the dependence of peripheral spike initiation on centrally generated bursting. To this end, we disconnected the STG from the peripheral nerves by cutting the *dvn*, bath applied DA, and stimulated the *dvn* (Figure 6A). We selected a burst stimulation pattern based on PD activity during unperturbed pyloric rhythms *in vitro* (Ballo and Bucher, 2009; Ballo et al., 2012). Bursts consisted of 19 stimuli with a parabolic interval structure, with a burst duty cycle of 0.35. We then varied the cycle period between 0.5 and 6 s. During normal pyloric activity, duty cycle and number of spikes in PD neurons do not change with cycle period across preparations (Bucher et al., 2005). Therefore, we changed burst duration proportionally to the cycle period and kept the number of stimuli per burst constant.

Recordings of the *pdn* during *dvn* burst stimulation at different periods showed that DA elicited prominent axonal spiking at longer cycle periods, but faster burst stimulation eliminated it (Figure 6B). Analysis of the dependence of mean peripheral spike rate on the period of PD burst stimulation showed that peripheral spike initiation was mostly eliminated at periods of 1 s or less, and still partially suppressed at cycle periods of several seconds (Figure 6C). In addition, the temporal structure of inhibition of peripheral spiking was dependent on the stimulus pattern. We analyzed the temporal distribution of peripheral spikes for stimulation periods of 2, 3, and 5 s by determining the percentage of spikes that occurred during the first second following a burst, in 100 ms bins (Figure 6D). At 2 s stimulation period, peripheral spiking was suppressed long enough that it increased throughout the whole 1s analysis window. This is consistent with an earlier observation that peripheral spikes during mixed activity predominantly occur in the second half of the interval between bursts (Bucher et al., 2003). However, at stimulation periods of 3 and 5 s, peripheral spiking was only suppressed in the first 100 ms bin after the end of a burst, and constant thereafter.

### Inhibition of Peripheral Spike Initiation by Centrally Generated Bursts Coincides With a Cumulative Slow After-Hyperpolarization

We wanted to test if the inhibition of peripheral spiking can be explained by subthreshold membrane potential changes in the axon. Peripheral spiking is due to an enhancement of the hyperpolarization-activated inward current (*I*_*h*_) by DA (Ballo et al., 2010). As the activation threshold of *I*_*h*_ is substantially more positive than the resting membrane potential, DA-mediated increase in *I*_*h*_ causes a depolarization of several millivolt in the quiescent axon, which is sufficient to reach spike threshold. Importantly, it also causes an increase in inward rectification, i.e., it counteracts activity-dependent hyperpolarization. In normal saline, bursting activity hyperpolarizes the axonal resting membrane potential, an effect that slowly accumulates over consecutive bursts and is likely caused by the Na^+^/K^+^ pump (Ballo and Bucher, 2009; Ballo et al., 2012; Zhang et al., 2017). In the presence of DA, when *I*_*h*_ is enhanced, this hyperpolarization is comparatively small. It is therefore reasonable to ask whether realistic bursting activity in the presence of DA can hyperpolarize the axon membrane potential enough to explain the inhibition of DA elicited peripheral spiking shown in Figure 6.

**FIGURE 7:**
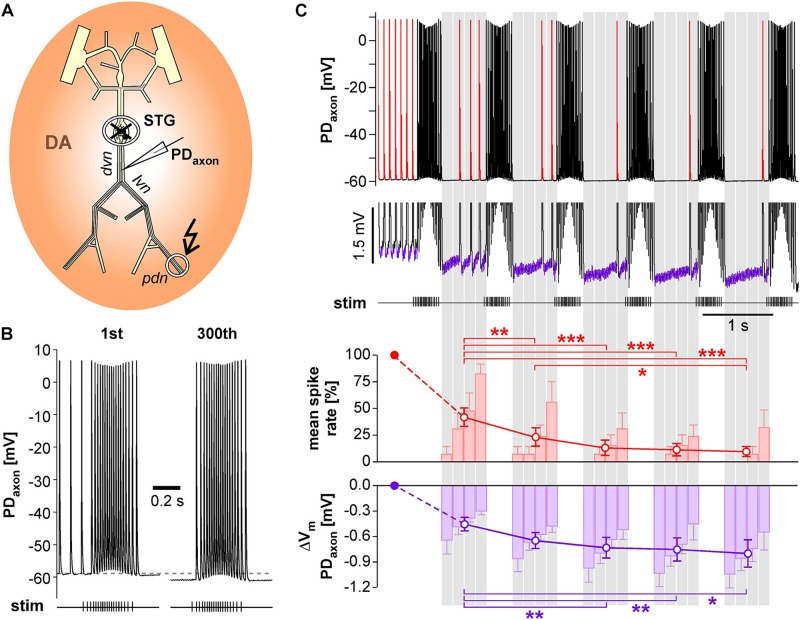
Intracellular PD axon recordings from the *dvn* show a cumulative after-hyperpolarization during the suppression of peripheral spike initiation. **(A)** Schematic of the STNS indicating the intracellular recording site in the *dvn*. Centrally generated activity was blocked and the *pdn* stimulated with a realistic burst pattern (kinked arrow), in the presence of DA. **(B)** Intracellular axon recording showing the moderate hyperpolarization of the resting membrane potentials over 5 min of burst stimulation at 1 Hz. Only the first and last burst are shown. **(C)** Example recording showing the suppression of DA elicited extraburst spiking in the first five interburst intervals (red spikes in the uppermost trace). To avoid unequal weighting across experiments with different spike rates, in each experiment, the spike rate in each interval was normalized to the mean spike rate before the first burst. Only experiments exhibiting peripheral spiking at least in the first interval between bursts were included (*n* = 6). After the initial drop, mean spike rate changed significantly across intervals (1W-RM-ANOVA, *p* < 0.001). Asterisks indicate significant differences in *post hoc* pairwise comparisons. Suppression of peripheral spiking was associated with a cumulative after-hyperpolarization across consecutive intervals (magnified voltage range in the middle trace, purple sections). After the initial drop, mean resting potential changed significantly across intervals (1W-RM-ANOVA, *p* < 0.001). Asterisks indicate significant differences in *post hoc* pairwise comparisons. To analyze temporal structure in the interval between bursts, data were averaged in 4 consecutive bins (gray shading). Spike rate increased across bins, while hyperpolarization decreased (RM-Rank-ANOVAs, *p* < 0.001 for both).

We performed intracellular axon recordings from PD close to the dominant peripheral spike initiation site in the *dvn* (Figure 7A). We then blocked centrally generated activity, applied DA, and stimulated the *pdn* with our realistic pattern at a burst frequency of 1 Hz. Intracellular PD axon recordings show overshooting spikes with little to no fast after-hyperpolarization, and slow enough repolarization to cause summation during bursts (Ballo and Bucher, 2009; Ballo et al., 2012; Figure 7B). Burst stimulation of the *pdn* confirmed that slow after-hyperpolarization in DA is modest even at steady state after 5 min (300 bursts; only first and last bursts shown). However, inhibition of peripheral spiking often happened immediately or after just a few cycles of bursting activity. Therefore, we specifically focused on hyperpolarization and peripheral spike initiation within the first five cycles of burst stimulation (Figure 7C). We only included recordings in which peripheral spiking was not completely abolished after the first burst, which was the case in 6 of 14 experiments. Across those experiments, the rate of extraburst spiking decreased to <10% within 5 cycles (red line plot in Figure 7C). This decrease in spiking was associated with a small cumulative hyperpolarization (<−0.8 mV) that followed a similar time course (purple line plot in Figure 7C). We also tested whether the time course of hyperpolarization matched the time course of peripheral spiking within the interval between bursts, as described in Figure 6D. Across experiments, peripheral spiking occurred predominantly in the later part of each interval (red bar plots in Figure 7C). This increase in spiking was associated with a partial recovery from hyperpolarization (purple bar plots in Figure 7C).

### The Balance Between *I*_*h*_-Mediated Depolarization and Na^+^/K^+^ Pump-Mediated After-Hyperpolarization in a Model Axon Determines the Activity-Dependence of Axonal Spike Initiation

**FIGURE 8:**
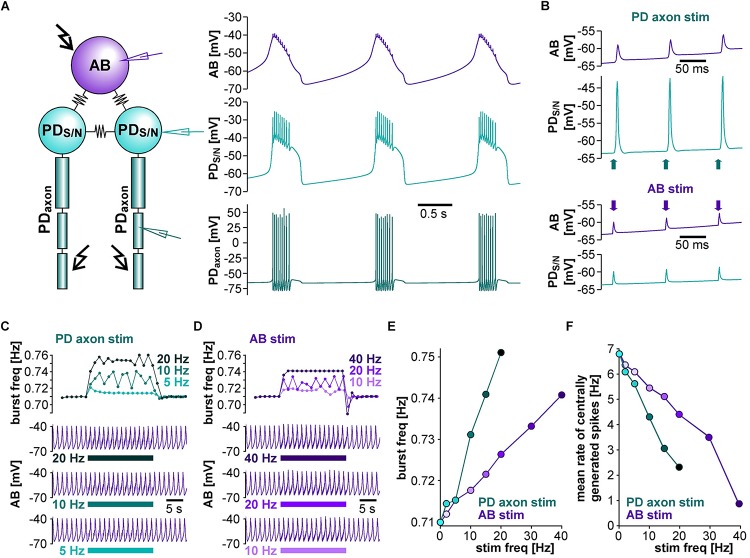
The effect of burst stimulation on spike initiation in the PD axon model. **(A)** Schematic of the 11-compartment axon model with its ionic currents, showing the recording site in the middle and the stimulation site (kinked arrow) at one end. **(B)** Stimulation of the tonically active axon at different burst frequencies (top to bottom: 0.33, 0.5, 0.67, and 1.0 Hz). Stimulation-elicited spikes are shown in gray. The stimulus pattern was the same as in the experimental protocol.

We used a modified version of our previously published model of the PD axon (Zhang et al., 2017; Figure 8A) to test whether its known ionic mechanisms are sufficient to give rise to the observed experimental results. In the model, we set *ḡ*_*h*_ to a value that produced tonic axonal spiking at 12 Hz, which is within the range of peripheral spike initiation in the PD axon when central activity is blocked and 1 μM DA is applied (Bucher et al., 2003). We then stimulated one end of the axon with the same burst patterns at different frequencies as shown in Figure 6. As in the biological axon, slow burst stimulation allowed for continuous axonal spike initiation during the interval between bursts, while spiking diminished with increasing burst frequency (Figure 8B). The model also replicated the experimental observation that at intermediate burst frequencies, spiking occurred predominantly in the later part of each interval (middle two traces in Figure 8B).

**FIGURE 9:**
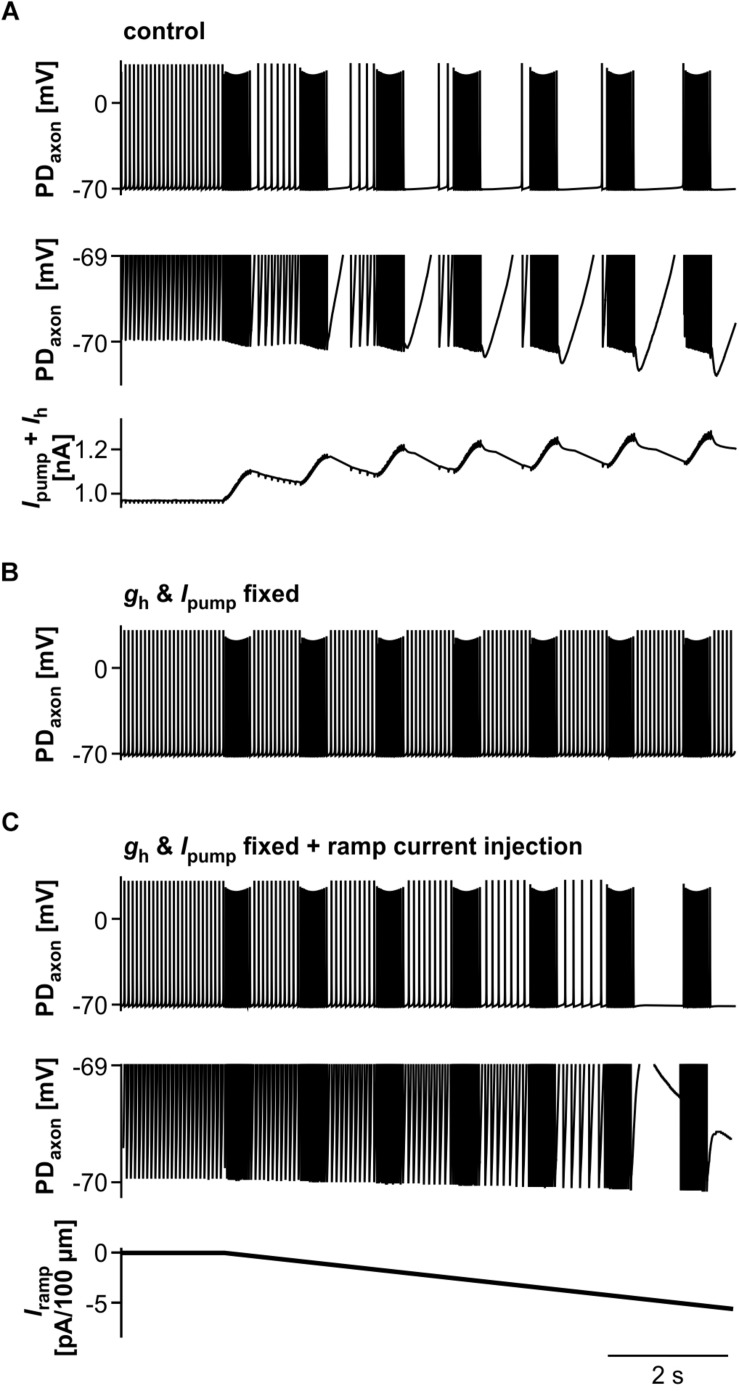
The role of *I*_*pump*_ and *I*_*h*_ in suppression of axonal spiking. **(A)** Burst stimulation of the axon at 0.67 Hz. Upper trace: Full voltage range. Middle trace: Expansion of the lower voltage region. Lower trace: Net outward current resulting from the sum of *I*_*h*_ and *I*_*pump*_. To exclude large deflections during spikes, all *I*_*h*_ values during voltages more depolarized than –69.8 mV were discarded. **(B)** Burst stimulation with *g*_*h*_ and *I*_*pump*_ fixed at their mean values before stimulation. **(C)** Hyperpolarizing current injection during burst stimulation, with *g*_*h*_ and *I*_*pump*_ fixed. Upper trace: Full voltage range. Middle trace: Expansion of the lower voltage region. Lower trace: Negative current ramp.

We then asked to which degree suppression of axonal spiking was associated with cumulative hyperpolarization, and whether the dynamic interactions between pump-mediated hyperpolarization and inward rectification through *I*_*h*_ were sufficient to explain the effect. As in the biological axon, cumulative hyperpolarization was present but in the sub-millivolt range (Figure 9A, top two panels). Underlying this hyperpolarization was the fact that inward rectification through *I*_*h*_ did not completely balance *I*_*pump*_, as the sum of both still yielded an outward current that built up across consecutive bursts (Figure 9A, lower panel). As in the biological axon, peripheral spike initiation occurred predominantly in the later part of the interval, associated with the partial recovery of outward current and hyperpolarization between bursts. To further test whether the dynamics of *I*_*pump*_ and *I*_*h*_ were necessary for the suppression of axonal spiking, we fixed *g*_*h*_ and *I*_*pump*_ at their mean values before stimulation. Under these conditions, burst stimulation did not suppress axonal spiking (Figure 9B). We then injected a negative ramp current into the axon with fixed *g*_*h*_ and *I*_*pump*_ values (Figure 9C). Again, baseline hyperpolarization in the sub-millivolt range was sufficient to suppress spiking. As the ramp current and resulting hyperpolarization increased continuously, without partial recovery between bursts, peripheral spike initiation decreased continuously.

## Discussion

### Interdependence of Different Spike Initiation Sites in the Same Neuron

We show here that in the PD neurons, central burst generation and distal axonal spike initiation in response to DA have mutually inhibitory effects. Different spike initiation sites in the same neuron can be functionally separated by external inputs, for example in CA3 pyramidal cells during gamma oscillations (Dugladze et al., 2012), but interactions across integration and initiation sites are more prevalent. One site can influence the other through subthreshold potentials only if both sites are electrotonically close to each other. In CA1 pyramidal cells, somatodendritic synaptic potentials do not decay completely over the first few hundred μm of axon length, and can therefore promote or suppress distal axonal spike initiation (Bahner et al., 2011; Thome et al., 2018). In the PD neurons, the electrotonic distance between central and peripheral spike initiation sites exceeds three length constants (Ballo and Bucher, 2009), and interactions must therefore be mediated by propagating spikes.

Spikes propagating from one site and invading the other can either suppress or promote spike initiation. Distal axonal spike initiation is promoted by highly repetitive spiking propagated from the proximal initiation site in hippocampal and cortical interneurons (Sheffield et al., 2011, 2013; Suzuki et al., 2014; Elgueta et al., 2015), as well as some stomatogastric motor neurons (Meyrand et al., 1992; Le et al., 2006). Suppression of spike initiation by invading spikes can exert functional dominance across different initiation sites in leech neurons (Calabrese, 1980; Maranto and Calabrese, 1983), an STG interneuron (Blitz and Nusbaum, 2008), and peripheral branches of C-fibers (Weidner et al., 2003). In some sensory neurons, peripheral spike initiation resulting from sensory integration can be partially suppressed by spikes backpropagating from central initiation sites (Pinault, 1995; Cattaert and Bevengut, 2002; Stadele et al., 2018).

While the examples above are well described at a phenomenological level, the cellular mechanisms underlying suppression of spike initiation by propagating spikes are not well understood. One possibility would be classical membrane refractoriness. In this case, spikes propagating from one initiation site to another would cause the membrane at the second site to become unexcitable or even extinguish spikes generated at the other site by collision (Gossard et al., 1999; Rossignol et al., 2006). However, the classic description of membrane refractoriness based on inactivation of Na^+^ channels and delayed deactivation of K^+^ channels only includes processes that occur at the time scale of a few milliseconds (Hodgkin and Huxley, 1952). Beyond the classical refractory period, excitability is usually enhanced (Krishnan et al., 2009; Bucher and Goaillard, 2011; Bucher, 2015). Consequently, suppression of activity at the other site would only be substantial at high spike frequencies, and spike collisions would only occur with high likelihood if the total propagation time from the first to the second initiation site exceeded the intervals of spikes generated at the first (Bucher et al., 2003; Daur et al., 2009). In the PD neuron, spike collisions during bursts have a very low likelihood, as the delay between STG and main peripheral spike initiation site in the *dvn* is only about 12 ms (Bucher et al., 2003), and extinction of peripherally generated spikes could only outlast each burst by these 12 ms. Therefore, suppression of activity by propagating spikes in the PD axon and elsewhere is more likely based on ionic mechanisms involving slower processes.

### Suppression of Central Bursting

Invading spikes could suppress activity by affecting the ability to generate spikes, or by interfering with the mechanisms underlying depolarization to threshold. For example, in CA1 pyramidal cells, backpropagating spikes can cause long-term depression of proximal synaptic potentials (Bukalo et al., 2013). The inhibitory effect of backpropagating PD spikes on slow pyloric pacemaker activity was due to a decrease in burst frequency and/or number of spikes per burst (Figure 2B). This was associated with a decrease in frequency and/or amplitude of slow wave depolarizations, which suggests that suppression of centrally generated spiking was mostly due to changes in subthreshold oscillatory behavior. Still, the decrease in the number of spikes per burst could have been either due to weaker oscillations or to a change in spike threshold or decrease in the ability to sustain repetitive spiking. However, antidromic spikes during sustained stimulation had stable amplitudes in soma recordings (Figures 2B,D,E), suggesting that at least there was no increase in refractoriness and the ability to generate spikes was robust at these stimulation frequencies.

AB is the only true oscillator in the pyloric circuit and crucial to rhythm generation (Miller and Selverston, 1982; Marder and Eisen, 1984; Marder and Bucher, 2007; Daur et al., 2016). Therefore, antidromic PD spikes likely do not just exert their effect through changes in PD excitability, but also have a substantial effect on AB’s oscillatory properties. Antidromic spikes invading the STG can fail to reach the sites of chemical synapses (Mulloney and Selverston, 1972), and electrical synapses are thought to be located at distal neurite branches (Cabirol-Pol et al., 2000; Rabbah et al., 2005). However, the prominent spikelets and even spiking responses in AB (Figure 2C) indicate that antidromic PD spikes are well transmitted through gap junctions. Pyloric cycle frequency can easily be manipulated by sustained current injections into AB, without much effect on burst duty cycle or activity phases of follower neurons (Sharp et al., 1993b; Hooper, 1997). As antidromic PD spikes are well transmitted to AB, the resultant net depolarization could explain the small to moderate increase in cycle frequency when modulatory input were intact and bursting otherwise robust (Figure 3).

We used a model of the pyloric pacemaker circuit to test whether the circuit structure and a fairly generic set of ionic conductances can replicate the effect of antidromic spiking on centrally generated activity. The model contained a simpler set of conductances than experimentally described and included in a prior model (Soto-Treviño et al., 2005) (see Materials and Methods), but produced activity with similar temporal features as the pyloric pacemaker, and allowed us to model different modulatory states that produced fast or slow activity. The latter was achieved by varying the modulator-activated inward current *I*_*MI*_, a low-threshold, non-inactivating, voltage-gated current elicited by a range of different neuromodulators, mostly neuropeptides. It is a potent activator of rhythmic activity and an important determinant of frequency in the pacemaker neurons (Sharp et al., 1993a, b; Swensen and Marder, 2000; Marder and Bucher, 2007; Zhao et al., 2010; Daur et al., 2016; Golowasch et al., 2017).

The model replicated both the robustness of fast burst generation (Figure 4) and the sensitivity of slow burst generation (Figure 5) to antidromic spiking. At a descriptive level, the suppression of slow bursting at lower values of *ḡ*MI was associated with a disruption of the slow trajectory of out-of-balance inward and outward currents. Complete cessation of bursting occurred when the consecutive intervals of antidromic spikes produced a fixed pattern of virtually identical current trajectories (Figure 5D). Thus, at low values of *ḡ*_*MI*_, each antidromic spike delayed the onset of subsequent burst, which, with sufficiently high stimulus frequency, resulted in an indefinite delay and the disruption of bursting.

A more mechanistic analysis of the underlying ionic mechanisms exceeded the scope of this study. Our model had reduced complexity, but neuronal slow wave oscillations are simple to generate and generally due to low-threshold regenerative inward currents that destabilize the resting state enough to slowly depolarize the cell, which in turn allows the activation of outward currents which return the voltage to more hyperpolarized values and restart the cycle (Bose et al., 2014; Golowasch et al., 2017). As such, oscillations only require a minimal set of inward and outward currents with the right kinetics and in the right quantitative balance, as little as a single inward and a single outward current in the generic Morris–Lecar model (Ermentrout and Terman, 2010). While the exact complement and the kinetics of currents determine the details of voltage trajectories, conductances interact in a highly non-linear fashion and can therefore only be tentatively mapped to specific membrane behavior (Taylor et al., 2009; Ermentrout and Terman, 2010). However, in general, for the transition from silent or tonic spiking to oscillatory behavior, the identity of currents matters less than the total balance of inward and outward currents (Amarillo et al., 2014; Golowasch et al., 2017). For example, pyloric pacemaker and follower neurons express the same type of currents, but follower neurons do not produce oscillations on their own due to higher levels of high-threshold K^+^ currents (Golowasch et al., 2017). For these reasons, it is more useful to functionally characterize oscillators according to their behavior, including responsiveness to perturbations (Izhikevich, 2000; Ermentrout and Terman, 2010). The fact that antidromic spiking could only disrupt slow bursting indicates that fast bursting activity in the same neurons obeyed a distinct phase resetting rule, and was therefore a qualitatively distinct type of bursting oscillator (Izhikevich, 2000). A mechanistic explanation of the behavior would therefore require a full mathematical analysis of burst disruption. However, minimally we can conclude that this behavior arises from common oscillator properties and does not depend on undescribed ionic mechanisms.

### Suppression of Peripheral Spike Initiation

The inhibitory effect of fast bursting on peripheral spike initiation outlasted each burst end by several hundred milliseconds (Figure 6), and could be due to a range of different phenomena. The PD axon exhibits a slow after-hyperpolarization that accumulates across consecutive bursts (Ballo and Bucher, 2009; Ballo et al., 2012). This is a common phenomenon in axons, often due to Na^+^/K^+^ ATPase activation (Baker, 2000; Kiernan et al., 2004; Moldovan and Krarup, 2006; Scuri et al., 2007; Bucher and Goaillard, 2011), and often counterbalanced by inward rectification through *I*_*h*_ (Grafe et al., 1997; Soleng et al., 2003; Baginskas et al., 2009; Bucher and Goaillard, 2011; Bucher, 2015). In the PD axon, DA increases *I*_*h*_ and consequently substantially reduces activity-dependent hyperpolarization, while blocking *I*_*h*_ increases it (Ballo et al., 2010, 2012). DA modulation of *I*_*h*_ is also the mechanism underlying peripheral spike initiation (Ballo and Bucher, 2009; Ballo et al., 2010). During quiescence, the threshold for spike initiation in the *dvn* is very close to the resting membrane potential, and DA/*I*_*h*_-mediated depolarization of less than 1 mV can elicit spikes (Ballo and Bucher, 2009). We show that fast burst stimulation eliminated peripheral spike initiation within a few cycles (Figure 7). Peripheral spiking appeared well correlated with baseline membrane potential, both within and across cycles. However, the total cumulative hyperpolarization over those few cycles was in the sub-millivolt range. The intracellular recording sites were close to the dominant site of peripheral spike initiation (Bucher et al., 2003), so measurements of membrane potential changes were unlikely to be subject to electrotonic decay.

We asked whether sub-millivolt hyperpolarization alone would be sufficient to suppress peripheral spike initiation. Small changes in membrane potential may have large effects on spike initiation due to an intricate balance between DA/*I*_*h*_-mediated depolarization, spike threshold, and slow after-hyperpolarization. Alternatively, there could be a decrease in excitability only indirectly or not at all related to changes in baseline membrane potential. One possibility is axonal shunting, that is, an activity- or modulator-dependent decrease in input resistance (Jackson and Zhang, 1995; Zhang and Jackson, 1995; Cattaert et al., 1999). Co-activation of opposing currents like *I*_*pump*_ and *I*_*h*_ is not accompanied by substantial changes in membrane potential, but can increase the total conductance level (Bucher and Goaillard, 2011). However, the increase in *g*_*h*_ with cumulative *I*_*pump*_ activation had a negligible effect on total membrane resistance of our axon model (not shown). Another possibility is a change in spike threshold caused by the ambiguous effects of slow changes in membrane potential. The immediate effect of hyperpolarization is that it moves the membrane potential away from the spike threshold, but more sustained hyperpolarization can dynamically change spike threshold and excitability by removing inactivation from both Na^+^ and A-type K^+^ channels (Debanne et al., 1999; De Col et al., 2008; Bucher and Goaillard, 2011; Bucher, 2015; Jiang et al., 2017). The PD axon expresses an A-type current and, as a result, spike amplitude and width are exquisitely sensitive to changes in membrane potential during repetitive activity (Ballo and Bucher, 2009; Ballo et al., 2012). We cannot exclude a contribution of shunting and spike threshold changes, but the results from our axon model suggest that the small hyperpolarization is important. The net cumulative outward current during sub-millivolt hyperpolarization was required for spike suppression (Figures 9A,B), and sub-millivolt hyperpolarization in the absence of changes in *I*_*pump*_ and *I*_*h*_ was sufficient to suppress axonal spike initiation without disrupting burst propagation (Figure 9C).

### Dynamic Regulation of the Relative Contributions of Two Spike Initiation Sites to Output Activity

An interesting aspect of our findings in the PD axons is that the suppressive effects of propagating spikes are bi-directional. Therefore, the contributions of each site to PD output activity can shift in an interdependent manner. During fast rhythmic pyloric activity, occurrence of peripheral spike initiation is unlikely. Resting hemolymph levels of biogenic amines are below 10 nM (Livingstone et al., 1980), and while in the absence of bursting activity the threshold for peripheral spike initiation is below 1 nM DA(Bucher et al., 2003), peripheral spiking is mostly extinguished even at 1 μM when cycle periods are ∼1 s or faster (Figure 6, Bucher et al., 2003). *In vitro* control pyloric periods are mostly between 1 and 2 s in *H. americanus* (Bucher et al., 2005, 2006). However, *in vivo* recordings in the closely related *H. gammarus* have shown that pyloric periods are longer than *in vitro*, and can increase to several seconds in the context of feeding and molting, and under hypoxic conditions (Clemens et al., 1998a, b, 1999, 2001). Experimentally, rhythmic activity can be slowed or interrupted by inhibitory neuromodulators (Cazalets et al., 1987, 1990; Pulver et al., 2003; Kwiatkowski et al., 2013). Under such conditions, peripheral spiking is likely to occur and can either produce tonic spiking output of PD, or give rise to mixed patterns in which bursts and lower frequency spiking alternate (Figures 1, 6). The relative contributions of each would then depend on the balance of the mutually inhibitory effects.

A similar interdependence has been described in a stomatogastric proprioceptor which has a peripheral spike initiation site that is activated by normal sensory transduction of in response to muscle stretch, and an additional central initiation site that is activated by neuromodulators and synaptic feedback from its target motor circuit (Daur et al., 2009; Stadele and Stein, 2016; Stadele et al., 2018). Activity generated at either site has specific distinguishable effects on central circuit operation, and their competitive interactions arising from mutual inhibition suggest that signal integration at either site can be dynamically adjusted. Therefore, interactions between proximal and distal spike initiation can shape both motor neuron output to stomach muscles and sensory feedback from muscle contractions. It would be interesting to assess the postsynaptic consequences of mixed PD activity at the target neuromuscular junctions, particularly because, in contrast to the sensory neuron, responses can be measured without the confound of recurrent connectivity. Crustacean stomach muscles are multi-terminally innervated and do not show fast Na^+^ spikes. However, synaptic responses can show substantial dynamics, due to combinations of different forms of short-term synaptic plasticity and non-linear muscle membrane properties (Lingle, 1981; Sen et al., 1996; Jorge-Rivera et al., 1998; Stein et al., 2006). In addition, stomach muscles have slow contraction properties that transform rhythmic input into mixtures of tonic baseline tension and phasic movements, integrating rhythmic input over multiple cycles (Morris and Hooper, 1998; Morris et al., 2000; Morris and Hooper, 2001). Therefore, the dynamic interactions between centrally and peripherally generated PD activity could play an important role in the production of movement.

## Data Availability Statement

The datasets generated for this study are available on request to the corresponding author.

## Author Contributions

ND, FN, and DB conceived and designed all experiments, and ND carried them out. YZ and FN conceived, designed, and carried out all modeling approaches. ND and DB analyzed the experimental data and generated the corresponding figures. FN analyzed the theoretical results and generated the corresponding figures. DB wrote the original draft of the manuscript. FN and ND critically reviewed and edited the text.

## Conflict of Interest

The authors declare that the research was conducted in the absence of any commercial or financial relationships that could be construed as a potential conflict of interest.

## Footnotes

1 http://neuron.yale.edu
